# Exploring the Impact of Access Cavity Designs on Canal Orifice Localization and Debris Presence: A Scoping Review

**DOI:** 10.1002/cre2.70013

**Published:** 2024-11-22

**Authors:** Mario Dioguardi, Davide La Notte, Diego Sovereto, Cristian Quarta, Andrea Ballini, Vito Crincoli, Riccardo Aiuto, Mario Alovisi, Angelo Martella, Lorenzo Lo Muzio

**Affiliations:** ^1^ Department of Clinical and Experimental Medicine University of Foggia Foggia Italy; ^2^ Department of Basic Medical Sciences, Neurosciences and Sensory Organs, Division of Complex Operating Unit of Dentistry “Aldo Moro” University of Bari Bari Italy; ^3^ Department of Biomedical, Surgical, and Dental Science University of Milan Milan Italy; ^4^ Department of Surgical Sciences, Dental School University of Turin Turin Italy; ^5^ DataLab, Department of Engineering for Innovation University of Salento Lecce Italy

**Keywords:** access cavity, ConsAC, endodontics, TradAC

## Abstract

**Objectives:**

One of the primary objectives in endodontics is to achieve thorough cleaning and disinfection of the root canal system during an endodontic procedure. This aims to reduce microbial contamination and prevent the development of endodontic lesions. To attain this goal, it is imperative to establish access to the endodontic space that allows for the complete removal of pulp tissue and the accurate identification of canal orifices while preserving the anatomical integrity of the root floor and pulp chamber as much as possible. In this scoping review, we aim to explore aspects related to the identification of canal orifices and the presence of pulp debris and residues during endodontic treatment. Specifically, we aim to assess whether and to what extent the design of the access cavity impacts these factors.

**Material and Methods:**

The scoping review was conducted and prepared following the Preferred Reporting Items for Systematic Reviews and Meta‐Analyses (PRISMA) guidelines (PRISMA Extension for Scoping Reviews [PRISMA‐ScR]).

**Results:**

The search yielded a total of 3697 bibliographic sources. After eliminating duplicates and applying eligibility criteria, only 10 studies were included.

**Conclusions:**

In conclusion, our review, conducted following PRISMA guidelines, includes 10 studies and suggests a potential trend: conservative techniques may generate more debris, whereas guided techniques exhibit superior precision in locating canal openings.

## 1 Introduction

One of the goals in endodontic treatment is to achieve thorough cleansing and disinfection of the root canal system to reduce microbial load and prevent the development of secondary endodontic lesions or the persistence of endodontic infections both inside and outside the roots (Neelakantan et al. 2017). To achieve predictable outcomes, it is imperative to obtain a tapered shaping of the canals, facilitating a three‐dimensional canal filling and ensuring complete disinfection. To accomplish this, it is crucial to attain access to the endodontic space that allows for the complete removal of pulp tissue and accurate identification of canal orifices, while endeavoring to preserve, as much as possible, the anatomy of the root floor and pulp chamber (Alovisi et al. 2022).

It is clear and evident that, in this context, proper planning and the selection of cavity design can influence the final outcome of endodontic treatment in terms of prognostic success (Fransson and Dawson 2023).

**Figure 1:**
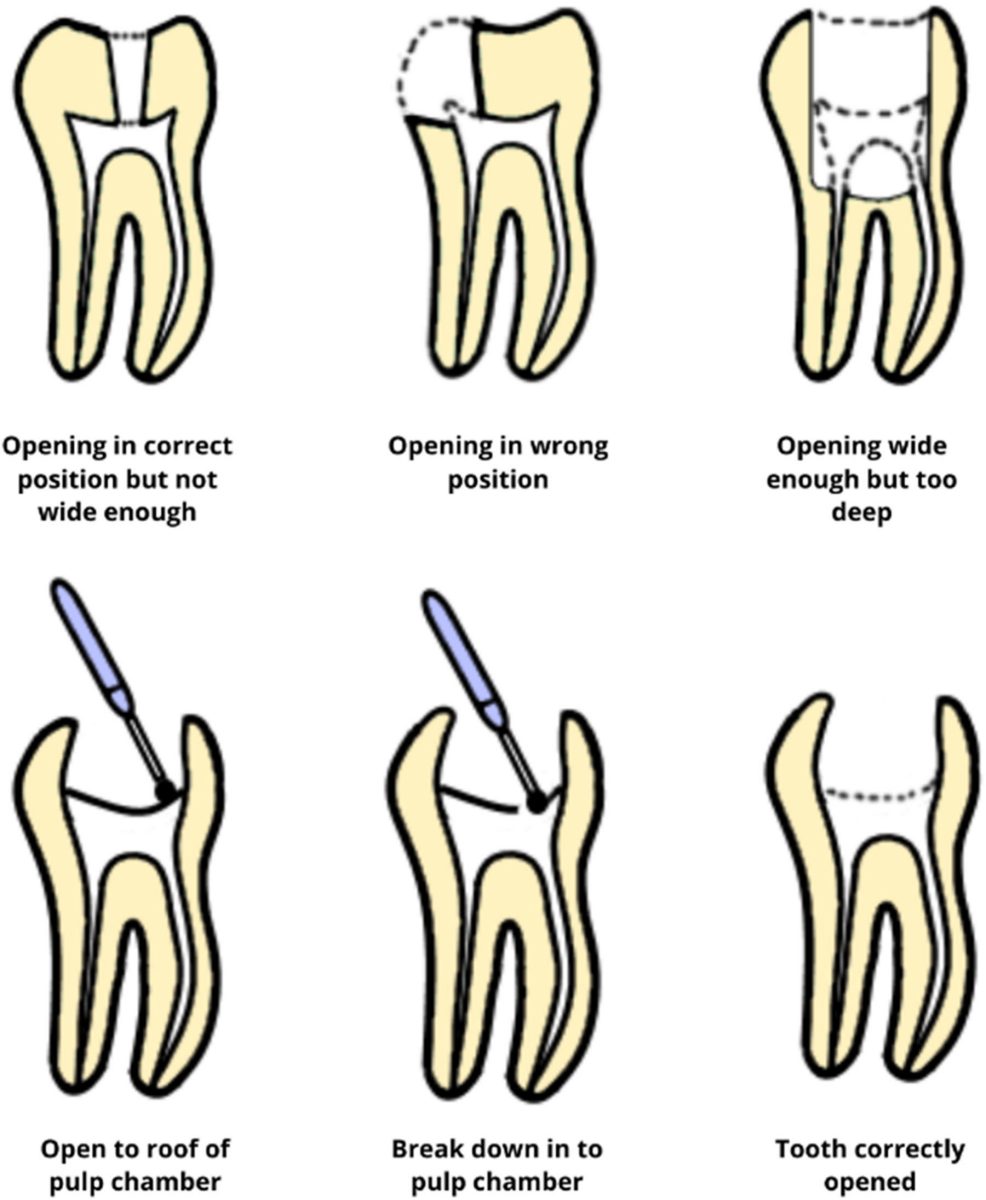
Images performed by Christian Quarta referring to the figures represented in Crane's book, *A Practicable Root‐Canal Technic*, which depict the incorrect procedures for opening the pulp chamber and the correct sequence for accessing the endodontic canals.

Among the earliest representations regarding the correct access cavity (AC) design to be used in the course of endodontic treatment, we find the description and illustrations through drawings performed by Crane in his 1920 book, *A Practicable Root‐Canal Technic* (Crane 1921), in which he describes techniques not to adopt, and the basic principles for a proper opening of the pulp chamber are already evident (Figure 1).

It is highlighted how Crane, in the past, already outlined all the principles of traditional pulp chamber preparation to achieve predictable success in endodontics. Indeed, he reported that the opening of the pulp chamber should provide direct access to each canal, aligned with its longitudinal axis while preserving the pulp chamber walls intact (Crane 1921).

Procedural errors in execution, as described by Crane, are currently quite common, especially among students (Almutairi et al. 2023) and young dentists undertaking initial endodontic treatments. Asymmetric openings with incomplete access to the pulp chamber and alterations to the chamber floor leading to perforations are frequent occurrences, as reported by Estrela et al. (2017).

The identification and teaching of traditional cavity preparation principles have enabled the standardization of cavity accesses, resulting in a reduction of errors made by dentists.

The principles of traditional access cavity (TradAC) preparation can be summarized in three fundamental points:

✓ Removal of the roof of the pulp chamber to expose the pulp horns.
✓ Creation of a smooth and unobstructed pathway to the canal orifices.
✓ Preservation of healthy dental structure.

Traditional approaches to endodontic cavity access, according to many authors, have led to an excessive sacrifice of dental tissue, reducing the resistance of the dental element (Mandil et al. 2022). Therefore, concurrently with traditional approaches, new cavity designs have emerged, some of which are defined as minimally invasive, with the aim of maximizing results with minimal sacrifice of dental tissue unaffected by pathology (Mookhtiar et al. 2019).

A recent literature review conducted by Mandil et al. (2022), which compares modern techniques with traditional approaches, concludes that some modern and conservative approaches are feasible options for canal treatment. They preserve the tooth structure, allowing for a quick and secure procedure (Mandil et al. 2022).

A clear and unambiguous classification of ACs still does not exist today. There is a lack of universally standardized and recognized taxonomy, partly due to the wide variety of AC designs. The literature reveals numerous nomenclatures, with terms and acronyms that often overlap or, conversely, resemble each other, even when referring to different cavity designs.

**Figure 2:**
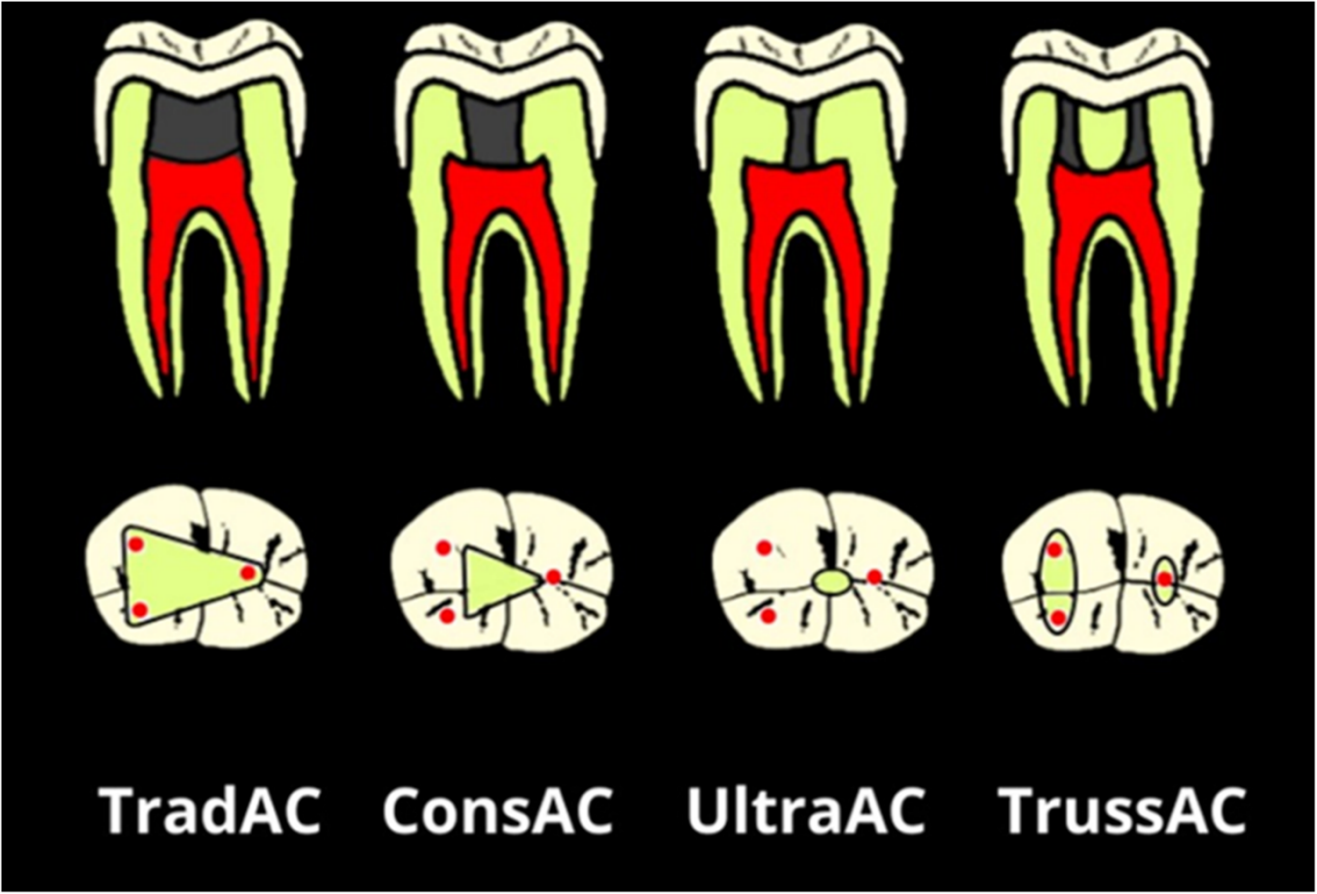
Representation of the access cavity design on the occlusal surface of the lower molar, upper molar, and upper premolar, using the UltraAC, TrussAC, ConsAC, and TradAC access techniques. Additionally, representation of the access cavities in a longitudinal section of the tooth. Images created by Christian Quarta based on the classification by Silva et al. (2020a, 2020b).

To reduce bias in this review, we will adopt the nomenclature proposed by Silva et al. (2020b), which categorizes cavity designs into six groups: TradAC, conservative access cavities (ConsAC), ultra‐conservative access cavities (UltraAC or NA [ninja access]), “Truss” access cavities (TrussAC), caries‐guided access cavities (CariesAC), and restoration‐guided access cavities (RestoAC) (Figure 2).

✓ TradAC: In posterior molars, the procedure involves removing the roof of the pulp chamber to allow straight‐line access to the canal orifices, with slightly converging axial walls, ensuring that all orifices are visible from the opening. When applied to anterior teeth, perpendicular access is achieved by removing the roof of the pulp chamber, pulp horns, and part of the lingual dentin, with further extension of the AC to the incisal edge.
✓ ConsAC: The goal of this approach is to minimize the removal of dentin tissue while keeping the roof of the pulp chamber intact. In posterior teeth, access begins from the central fossa of the occlusal surface and proceeds with the removal of dentin tissue until the canal orifices are identified. The axial walls converge slightly toward the occlusal surface, aiming to preserve the roof of the chamber to the maximum. This type of access can also be achieved with divergent walls. In anterior sectors, the opening is located more on the lingual or palatal surface, creating a small triangular or oval cavity to preserve both pulp horns and a greater amount of pericervical dentin.
✓ UltraAC: This pulp chamber opening is similar to the one described earlier but without requiring further extensions to preserve the roof of the pulp chamber as much as possible. In anterior sectors, the opening can be made at the center of the incisal edge, parallel to the longitudinal axis of the tooth. This type of access is also known as “ninja access.”
✓ TrussAC: In multi‐rooted teeth, the goal is to maintain a tooth‐dentin bridge between the ACs made on the occlusal surface perpendicular to the individual canal orifices.
✓ CariesAC: The opening is performed by only removing caries (if present), while preserving all remaining enamel–dentin tissue.
✓ Restoration‐guided cavity (RestoAC): The opening is performed by removing only the restoration (if caries is not present), while preserving all remaining enamel–dentin tissue.

Some operational strategies in the therapeutic approach, such as the use of operative microscopy in dentistry, have indeed allowed endodontic treatment to become more conservative and predictable. This enables greater precision in the execution of the AC, regardless of its design.

The design of the AC plays a crucial role in endodontic treatment. Conservative techniques, such as the ConsAC and TrussAC, are performed to preserve the maximum amount of coronal dentine and enamel. This preservation aims to enhance the fracture resistance of dental elements, which, for strategic prosthetic reasons, must endure significant masticatory loads. The TrussAC, in particular, involves creating openings in the chamber roof only at the level of the orifices. This approach necessitates meticulous planning, which is greatly facilitated by the use of cone‐beam computed tomography (CBCT) and the design of guided access openings.

In the context of conservative access openings, the teeth that stand to benefit the most from careful design are undoubtedly the upper and lower molars. Additionally, any clinical situation involving multi‐rooted teeth with atypical root anatomy would benefit significantly from such planning.

Concurrently, CBCT, the use of which is increasingly recommended in recent guidelines for endodontics (Chugal et al. 2023), allows the detection of extra canals and complex anatomical variations during the diagnostic phase (Patel et al. 2014).

Furthermore, there has been an improvement in endodontic instruments, which are increasingly capable of withstanding stress without fracturing or deviating from the canal's anatomy (Pawar and Singh 2021).

Advancements have also focused on the activation of irrigants, allowing for the disinfection and cleansing of areas within the canal system that would otherwise be inaccessible, without requiring excessive enlargement of the root canal during the shaping phase.

Considering these operational factors, such as the use of optical microscopy and irrigant activation, along with diagnostic factors, such as CBCT, raises the question of whether conservative or minimally invasive approaches, as suggested by Clark and Khademi, may partially compromise one or more of the objectives of endodontic treatment. In fact, in a recent article, Clark and Khademi argue that a traditional access approach must strike a balance between three fundamental aspects: the operational needs of the endodontist, the remaining dental structures, and the achievement of an apical‐coronal seal that minimizes the risk of recurrence (Clark and Khademi 2010).

Clark and Khademi believe that traditional access does not always adequately respect the remaining coronal structures, a condition that can lead to medium to long‐term failures due to the loss of the coronal seal through fracture or functional unusability of the treated tooth, thus promoting the adoption of more conservative pulp chamber accesses (Clark and Khademi 2010).

From another perspective, TradAC, which involves removing the roof of the pulp chamber and the protrusions of cervical dentin while widening the canal orifice, ensures greater ease in locating and probing all orifices of the root canals, providing direct access to the apical foramen. This could lead to a reduction in issues related to the shaping, cleaning, and filling phases. Moreover, perpendicular access to the canal would facilitate the unobstructed insertion of rotary instruments, thereby reducing the risk of fatigue fractures.

In certain clinical situations, the implementation of conservative access openings such as TrussAC or ultraAC is a subject of controversy. Although preserving portions of the chamber roof provides better fracture resistance against occlusal loads, concerns have arisen regarding unprepared areas that may become colonized by bacteria, leading to biofilm formation (Alovisi et al. 2018). Additionally, many studies have reported the presence of debris. Conversely, studies such as those by Rover et al. (2020) and Silva et al. (2020a) have indicated that unprepared areas resulting from endodontic instrumentation, as well as orifice localization as reported by Mendes et al. (2020), yield comparable outcomes across different AC designs. Given the increased technical difficulty in performing these new conservative access designs, the actual benefit in terms of predictability and long‐term prognosis of endodontic treatment, whether traditional or conservative, must be carefully evaluated.

Improper planning and execution of the pulp chamber AC can lead to alterations of the floor, resulting in changes to the position of the canal orifice within the context of the floor, making it increasingly difficult to probe and locate the canal effectively. For example, conservative techniques such as TrussAC, while providing access to the pulp chamber directly above the canal orifices, can result in areas of the pulp chamber roof that are inadequately cleaned. This promotes the accumulation of procedural debris and necrotic tissue that is not properly removed. In contrast, with TradAC, the pulp chamber roof is completely removed, providing direct access to the canal orifices, which are appropriately enlarged (Memiş and Karataş 2023, 2022).

The foundation of conservative access approaches lies in the concept that preserving pericervical dentin helps protect endodontically treated teeth from fracture. Most fractures tend to occur not only at the cuspal level but also at the pulp chamber floor. Therefore, it follows that the less stress placed on the pericervical dentin results in a lower risk of post‐treatment fractures. Studies indicate that TradAC results in the greatest loss of dentinal tissue (Shabbir et al. 2021; Maske et al. 2021).

However, it is not yet clear how the design of the AC influences mechanical stress, which is a significant disadvantage of TradAC due to the associated fractures (Maske et al. 2021). Studies report that TradAC overall shows a higher incidence of cuspal chipping patterns and a greater number of irreparable fractures (Makati et al. 2018). One explanation might be that increasing the size of the endodontic cavity preparation maximizes cusp deflection during restorative procedures, thereby promoting fracture (Mowlood, Ali, and Mahdee 2022).

Minimally invasive access procedures are advantageous primarily in terms of fracture resistance, reducing the risk of post‐endodontic fractures (Motiwala, Gul, and Ghafoor 2022). Teeth benefit from improved mechanical stability and greater long‐term functional survival. Additionally, certain access techniques, such as TrussAC and guided access procedures using CBCT, provide direct access to the canal orifices while preserving canal tissue (Long et al. 2023). Post‐endodontic restorations are also less complex and destructive, resulting in reduced costs (Ballester et al. 2021).

The main objective of this study is to provide essential information to clinicians regarding AC designs, aiming to present all available scientific literature data concerning issues related to the localization of canal orifices and the presence of pulp and dentin residues and debris within endodontic preparations in relation to ACs. The primary outcome focuses on the impact of AC designs on the localization of canal orifices in the pulp chamber. The secondary outcome aims to assess the effect of the presence of debris in relation to AC designs.

Therefore, in this scoping review, we have examined aspects related to the identification of canal orifices and the presence of pulp debris and residues during endodontic treatment to assess whether and to what extent the design of the AC may impact these factors, ultimately influencing endodontic prognosis.

## 2 Materials and Methods

### 2.1 Protocol and Registration

This scoping review was written and performed following the Preferred Reporting Items for Systematic Reviews and Meta‐Analyses (PRISMA) Extension for Scoping Reviews (PRISMA‐ScR) checklist as reported by Tricco et al. (2018). Although the review protocol was prepared before the database search was executed, it was deemed appropriate not to register it.

The decision not to register the protocol, made in agreement with all reviewers, is based on the fact that in the International Prospective Register of Systematic Reviews (PROSPERO), it is not possible to register scoping reviews. Transforming the current scoping review into a systematic review would have been a deviation from the previously established protocol, betraying the a priori established research methodology that envisioned the execution of a scoping review.

### 2.2 Eligibility Criteria

All studies examining ACs in endodontics concerning the identification of canal orifices and the presence of pulp debris and residues during endodontic treatment were considered potentially eligible. No restrictions were imposed based on the year of publication or language, as long as an English abstract was available. Literature reviews were excluded and solely utilized for bibliographic research and gaining additional insights into the review topic.

### 2.3 Information Sources

The search was conducted across three databases (PubMed, Scopus, and ScienceDirect) and a registry (Cochrane Library). Additionally, a gray literature search was performed on Google Scholar and Opengray (DANS EASY Archive).

The inclusion of gray literature sources aims to minimize publication bias. Often, scientific studies reporting nonstatistically significant data when comparing two methodologies (in this case, for cavity access) might not be published due to the lack of scientific relevance of the data in indexed and high‐impact journals. However, this data may be found in studies presented at conferences, in preprints, or in doctoral theses—sources typically absent from major databases. This information is crucial when conducting a meta‐analysis to avoid distortions in aggregated results. Relying solely on significant studies could introduce bias, directing the final effect toward a specific treatment compared to the control.

Potentially eligible articles were also sought among the references of literature reviews related to ACs in endodontics.

Systematic reviews and literature reviews were excluded from the process of identification and selection of studies to be included, which focused exclusively on primary research studies.

The research was carried out from October 10, 2023 to November 1, 2023, with the last update of identified records completed on November 3, 2023.

### 2.4 Search

The authors responsible for researching the studies used the following keywords in the databases: TradAC OR ConsAC OR UltraAC OR TrussAC OR access cavity design endodontic.

The keywords used on PubMed are shown below:

Search: TradAC OR ConsAC OR UltraAC OR TrussAC OR access cavity design endodontic (sort by: Most Recent).

“TradAC”[All Fields] OR “ConsAC”[All Fields] OR “UltraAC”[All Fields] OR “TrussAC”[All Fields] OR ((“access”[All Fields] OR “accessed”[All Fields] OR “accesses”[All Fields] OR “accessibilities”[All Fields] OR “accessibility”[All Fields] OR “accessible”[All Fields] OR “accessing”[All Fields]) AND (“cavity s”[All Fields] OR “dental caries”[MeSH Terms] OR (“dental”[All Fields] AND “caries”[All Fields]) OR “dental caries”[All Fields] OR “cavities”[All Fields] OR “cavity”[All Fields]) AND (“design”[All Fields] OR “design s”[All Fields] OR “designabilities”[All Fields] OR “designability”[All Fields] OR “designable”[All Fields] OR “designed”[All Fields] OR “designer”[All Fields] OR “designer s”[All Fields] OR “designers”[All Fields] OR “designing”[All Fields] OR “designs”[All Fields]) AND (“endodontal”[All Fields] OR “endodontic”[All Fields] OR “endodontical”[All Fields] OR “endodontically”[All Fields] OR “endodontics”[MeSH Terms] OR “endodontics”[All Fields])).

Translations access: “access”[All Fields] OR “accessed”[All Fields] OR “accesses”[All Fields] OR “accessibilities”[All Fields] OR “accessibility”[All Fields] OR “accessible”[All Fields] OR “accessing”[All Fields]; cavity: “cavity's”[All Fields] OR “dental caries”[MeSH Terms] OR (“dental”[All Fields] AND “caries”[All Fields]) OR “dental caries”[All Fields] OR “cavities”[All Fields] OR “cavity”[All Fields]; design: “design”[All Fields] OR “design's”[All Fields] OR “designabilities”[All Fields] OR “designability”[All Fields] OR “designable”[All Fields] OR “designed”[All Fields] OR “designer”[All Fields] OR “designer's”[All Fields] OR “designers”[All Fields] OR “designing”[All Fields] OR “designs”[All Fields]; endodontic: “endodontal”[All Fields] OR “endodontic”[All Fields] OR “endodontical”[All Fields] OR “endodontically”[All Fields] OR “endodontics”[MeSH Terms] OR “endodontics”[All Fields].

On December 18, 2023, a search of records on PubMed was conducted to update the records, incorporating additional keywords to include more studies.

Search: access cavity endodontic.

(“access”[All Fields] OR “accessed”[All Fields] OR “accesses”[All Fields] OR “accessibilities”[All Fields] OR “accessibility”[All Fields] OR “accessible”[All Fields] OR “accessing”[All Fields]) AND (“cavity s”[All Fields] OR “dental caries”[MeSH Terms] OR (“dental”[All Fields] AND “caries”[All Fields]) OR “dental caries”[All Fields] OR “cavities”[All Fields] OR “cavity”[All Fields]) AND (“endodontal”[All Fields] OR “endodontic”[All Fields] OR “endodontical”[All Fields] OR “endodontically”[All Fields] OR “endodontics”[MeSH Terms] OR “endodontics”[All Fields]).

Translations access: “access”[All Fields] OR “accessed”[All Fields] OR “accesses”[All Fields] OR “accessibilities”[All Fields] OR “accessibility”[All Fields] OR “accessible”[All Fields] OR “accessing”[All Fields].

Cavity: “cavity's”[All Fields] OR “dental caries”[MeSH Terms] OR (“dental”[All Fields] AND “caries”[All Fields]) OR “dental caries”[All Fields] OR “cavities”[All Fields] OR “cavity”[All Fields].

Endodontic: “endodontal”[All Fields] OR “endodontic”[All Fields] OR “endodontical”[All Fields] OR “endodontically”[All Fields] OR “endodontics”[MeSH Terms] OR “endodontics”[All Fields].

### 2.5 Selection of Sources of Evidence

The search for eligible articles and reports was conducted by two reviewers (M.D. and D.L.N.), with a third reviewer (C.Q.) responsible for deciding whether to include studies in case of conflicts. The two reviewers reached a consensus on eligibility criteria, keywords, and selected databases. They independently conducted the search for articles and reports and recorded the number of articles obtained for each keyword and from each database. Duplicate studies from different databases were removed using EndNote 9 software (Philadelphia, PA, USA), and any overlapping studies not compatible with EndNote were manually excluded during the screening phase. The two reviewers then proceeded to screen and include studies, with discussions and debates on which studies to include.

### 2.6 Data Charting Process, Data Items, Synthesis of Results, Risk of Bias

The characteristics and the data to be extracted from the studies were determined collaboratively by the two reviewers immediately after the study selection phase. The extracted data included the first author, year of publication, bibliographic reference, study type, the specific cavity access design investigated, the number of samples or teeth tested, and the main results or conclusions of this study. This data extraction process was carried out independently by the two reviewers, each using separate tables. Subsequently, a third reviewer cross‐verified the accurate entry of data.

The risk of bias was evaluated using the Checklist for Reporting In Vitro Studies (CRIS) guidelines, proposed for the assessment of in vitro dental studies.

## 3 Results

### 3.1 Results of Sources of Evidence

The search in Science Direct Library (3146), SCOPUS (186), PubMed (305), and Cochrane Library (60) produced a number of bibliographic sources equal to 3697. With the elimination of duplicates and the elimination of studies not related to the topic of review, we identified a number of potentially eligible articles. From these, 30 were considered, but only 10 fully met the eligibility criteria and were included in the quantitative assessment.

**Figure 3:**
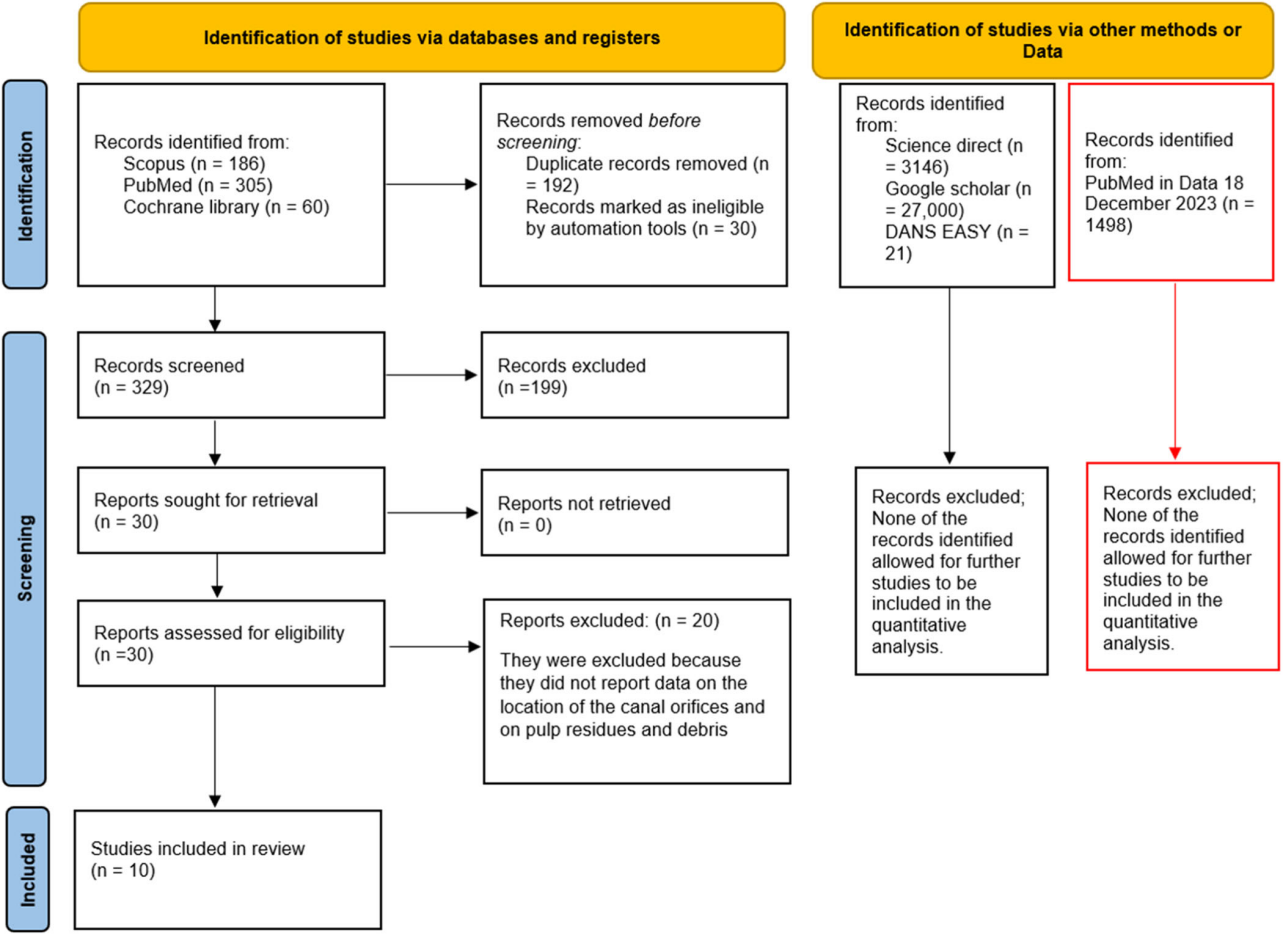
Entire selection and screening procedures are described in the PRISMA flowchart. The red boxes show the results of the research conducted on December 18 on PubMed using the keyword “access cavity endodontic.”

Furthermore, the gray literature analysis (http://www.opengrey.eu, DANS EASY Archive, and Google Scholar) and previous systematic reviews did not allow for the identification of additional studies to be included in the quantitative assessment. The update of records using the keyword “access cavity endodontic” conducted on PubMed on December 18, 2023, yielded a total of 1498 records. Screening these additional records did not result in the inclusion of further studies that had already been considered. The entire procedure of the identification, selection, and inclusion of the studies is indicated in the flowchart of Figure 3.

### 3.2 Characteristics of Sources of Evidence and Results of Individual Sources of Evidence

A total of 10 studies were included in the scoping review, categorized into two primary outcomes.

For the first outcome, concerning the localization of canal orifices in the pulp chamber, seven studies were incorporated (Table 1): Dianat et al. (2020), Jain et al. (2020), Rover et al. (2017), Saygili et al. (2018), Mendes et al. (2020), Connert et al. (2019), and Buchgreitz, Buchgreitz, and Bjørndal (2019).

**Table 1:** Data extracted from the seven studies included, the number of trucks tested, the access cavity design, and the main results reported: LAC (standard lingual access cavity), TradAC (traditional access cavity), DNA (dynamic navigator access), IAC (incisal access cavity), NA (ninja access), CAC ConsAC (conservative access cavity), GAC (guided access cavity), and TrussAC (truss access cavities).

Reference	Country	Teeth	Design access cavity	Results
Dianat et al. (2020)	USA	60 single‐rooted teeth	LAC, TradAC, DNA	No significant difference in the number of unsuccessful attempts to locate calcified canals
Jain et al. (2020)	USA	40 maxillary and mandibular central incisors (3D printed to simulate canal calcification)	IAC, DNA	Increased precision in the DNA group for locating calcified canals
Rover et al. (2017)	Brazil	30 extracted intact maxillary first molars	TradAC	Increased canal detection in the TradAC group with and without magnification
Saygili et al. (2018)	Turkey	60 roots of extracted human maxillary first molars	TradAC, ConsAC, NA	ConsAC and TradAC had significantly more MB2 detection compared with NA
Mendes et al. (2020)	Brazil	60 extracted mandibular first molars	TradAC, NA	No significant difference in the detection of the middle mesial canal between the groups
Connert et al. (2019)	Switzerland, Germany	Six models (three upper and three lower jaws)	LAC, GAC	Increased canal location in GAC compared with LAC
Buchgreitz, Buchgreitz, and Bjørndal (2019)		44 of the teeth	GAC	Clinical implementation of the guide resulted in precise localization and negotiation of the root canal

For the second outcome, which pertains to pulp residues and debris, four studies were identified (Table 2): Silva et al. (2020a), Rover et al. (2020, 2017), and Neelakantan et al. (2018).

**Table 2:** Data extracted from the four studies included (remaining pulp tissue and hard tissue debris).

Reference	Country	Teeth	Design access cavity	Results
Silva et al. (2020a)	Brazil	52 extracted two‐rooted maxillary premolars	TradAC, NA	NA had more hard tissue debris compared with TradAC
Rover et al. (2020)	Brazil	40 extracted mandibular incisors	LAC, IAC‐C	No difference in accumulated hard tissue debris
Rover et al. (2017)	Brazil	49 extracted first maxillary molars extracted	TradAC, ConsAC	No significant difference
Neelakantan et al. (2018)	China	24 extracted mandibular first molar	TradAC, TrussAC	The remaining pulpal tissue in the pulp chamber was significantly more in Truss compared with TradAC; no significant difference in residual pulpal tissue in root canals and isthmuses

One study, by Rover et al. (2017), was included for both outcomes.

For the first outcome, approximately 399 samples were utilized, including extracted teeth and printed resin models, in addition to six models (three upper and three lower jaws). In the case of the second outcome, 165 samples were subjected to testing.

TradAC was the most frequently evaluated method (six studies), followed by NA and standard lingual access cavity (LAC), each with three studies. All the extracted data are presented in Tables 1 and 2.

However, to conduct a meta‐analysis of the data, significant heterogeneity was observed in the teeth, samples, and the cavitary access designs used.

### 3.3 Risk of Bias

The risk of bias was assessed based on the CRIS guidelines (Krithikadatta, Datta, and Gopikrishna 2014) proposed to evaluate in vitro dental studies. The results are shown in Table 3. For each question in the risk of bias study, a value from 1 to 5 was assigned (where 1 = high risk and 5 = low risk). The questions that the reviewers answered by assigning a score were as follows:

1. For the sample size calculation, “Is the sample size adequate for obtaining statistically significant results?”
2. For meaningful difference between groups, “Has the 'meaningful difference' measurement been set correctly in the groups taking into account the sample size and the type of measurement?”
3. For sample preparation and handling, “Does the study describe information on the production or handling of the samples to be tested?”
4. For allocation sequence, randomization, and blinding, “Did the samples have equal and independent possibility of a sample entering any group?”
5. For statistical analysis, “Are the statistical methods described?”

**Table 3:** Assessment of the risk of bias within the studies, with scores 7–12 = low quality, 13–20 = intermediate quality, and 21–25 = high quality.

Reference	Sample size calculation	Meaningful difference between groups	Sample preparation and handling	Allocation sequence, randomization, and blinding	Statistical analysis	Score
Dianat et al. (2020)	4	5	5	5	4	23
Jain et al. (2020)	4	5	4	4	4	21
Rover et al. (2017)	5	5	5	3	4	22
Saygili et al. (2018)	4	5	5	3	4	21
Mendes et al. (2020)	5	5	5	4	4	23
Connert et al. (2019)	4	5	5	3	4	21
Silva et al. (2020a)	5	5	5	3	4	22
Rover et al. (2020)	3	5	5	5	4	22
Neelakantan et al. (2018)	3	5	5	4	4	21

Studies presenting a high risk of bias were not included in the scoping review and were eliminated during the inclusion phase and some during the data extraction phase (Table 3). The risk of bias assessment of the five included articles was conducted by M.D. and A.B.

The study by Buchgreitz, Buchgreitz, and Bjørndal (2019) was excluded from the risk of bias assessment because it refers to a clinical trial rather than an in vitro study. Given the nature of scoping review which is not a systematic review, it was decided to proceed with data extraction anyway.

## 4 Discussion

This scoping review aimed to summarize all the results obtained concerning the influence of the AC design to the pulp chamber on the localization of canal orifices, and, secondarily, on the presence of remaining pulp residues and debris following the shaping of endodontic canals. This review encompassed a total of only 10 studies, involving around 565 tested samples.

### 4.1 Canal Orifice

One of the fundamental aspects for achieving success in endodontics is the identification of all canal orifices, including those that are challenging to locate, such as the MB2 (second mesiobuccal canal in maxillary first molars) and the MMC (mesial middle canal in mandibular first molars).

In a systematic review conducted by Shabbir et al., which focused on guided cavity access, three studies exclusively examining non‐guided opening techniques were reviewed (Dianat et al. 2020; Jain et al. 2020). Of these, only one showed easier canal localization in teeth accessed using TradAC compared to ConsAC (Shabbir et al. 2021).

However, in the single study where TrussAC was also employed (Saber et al. 2020), it was found that this approach posed greater challenges for operators in detecting the second mesiobuccal canal compared to ConsAC and TradAC.

In another systematic review conducted by Ballester et al., three studies were examined (Rover et al. 2017; Saygili et al. 2018), with two focusing on locating the MB2 canal in maxillary first molars and one on detecting the MMC canal in mandibular first molars (Mendes et al. 2020), all without the use of optical magnification tools.

This review revealed that TradAC yielded a higher success rate in locating MB2 canals compared to ConsAC, in contrast to the findings of the previously mentioned systematic review (Ballester et al. 2021).

However, when utilizing the operating microscope, no significant differences were observed between TradAC and ConsAC. Regarding UltraAC, it was noteworthy that significantly fewer MB2 canal localizations were achieved compared to TradAC, aligning with the findings reported in Shabbir et al.'s (2021) review, even in cases where the operating microscope was employed.

As for the MMC canal, no differences were observed between ConsAC and TradAC, both with and without the assistance of the operating microscope. The operating microscope demonstrated superior canal localization capabilities for both access techniques (TradAC and ConsAC).

To gather additional insights into the UltraAC access, this review included an in vitro study focused on the localization of the MB2 canal, conducted by Saygili et al. In the article under consideration, a sample of 60 maxillary molars was analyzed, where access preparations were performed using the TradAC, ConsAC, and UltraAC techniques, all with the assistance of the operating microscope (Saygili et al. 2018).

The results revealed that no statistically significant differences were found between TradAC and ConsAC. However, the UltraAC technique exhibited a statistically lower MB2 canal detection rate (Saygili et al. 2018). This difference is particularly relevant, especially when considering that all available tools were used in the study, except for preliminary CBCT, to facilitate canal localization, including the operating microscope, ultrasonic preparation, and pre‐flaring.

The article mentioned suggests that these already statistically significant differences may further intensify in cases where such tools are not utilized.

The authors emphasize that in TrussAC, where the localization of the MB2 canal is more challenging, it is likely to increase the risk of complications. These complications may include instrument fractures and canal transportation, primarily due to the absence of straight‐line access for instrument entry into the canals.

In a recent systematic review conducted by Kapetanaki et al., in alignment with the previously discussed results (Buchgreitz, Buchgreitz, and Bjørndal 2019; Gluskin, Peters, and Peters 2014), it is asserted that reducing the dimensions of ACs leads to a higher risk of failing to detect specific radicular canals (Kapetanaki, Dimopoulos, and Gogos 2021).

From this, it can be inferred that it is still uncertain whether the ConsAC, when performed without the assistance of a microscope, poses an obstacle for operators in locating canal orifices.

This scenario does not apply to miniaturized access cavities (MiniAC), where studies demonstrate a greater challenge in canal identification, whether or not the intraoperative microscope is utilized (Addy 2000).

### 4.2 Pulp Residues and Debris

Many systematic reviews have focused on quantifying the volume of residual hard tissue debris within the root canals, resulting from canal shaping.

In the review by Shabbir et al., three articles were included (Rover et al. 2020; Silva et al. 2020a) to analyze this parameter, and none of them demonstrated statistically significant differences in the number of dentinal debris in the root canals based on the different AC techniques applied to the samples.

Similar results were found in the review by Ballester et al., only when comparing TradAC and ConsAC. However, the UltraAC showed a statistically higher quantity of dentinal debris following canal shaping (Ballester et al. 2021).

It can be concluded that current data are still insufficient to determine whether different AC techniques influence the amount of dentinal residues from canal shaping, which could hinder proper irrigation and disinfection of the root canals.

The quantity of residual pulp tissue after canal shaping, located in both the pulp chamber and the root canals, exhibited varying outcomes when using two different access techniques, specifically, TrussAC and TradAC, as documented by Neelakantan et al. (2018). In particular, TrussAC resulted in a greater volume of remaining pulp tissue exclusively within the pulp chamber, whereas no statistically significant differences were observed within the root canal.

These data are consistent with the findings of Silva et al. (2020a), regarding residual dentinal tissues, which showed a higher occurrence with minimally invasive techniques.

### 4.3 Summary Evidence

Openings performed with traditional accesses (TradAC) have demonstrated a greater ability to locate canal orifices compared to more conservative methods, such as ultra access (NA), especially when coupled with intraoperative optical microscopy. The only study among those included that reports data without significant differences is the one conducted by Mendes et al. (2020). Additionally, methods involving canal guidance through the use of CBCT and guides have shown a greater localization of canal orifices.

Furthermore, it is evident from the included studies that there is a higher presence of debris and pulp residues in more conservative methods, such as Truss and NA. The only study reporting data not aligned with the observations of other authors is that of Rover et al. (2017), which does not detect statistically significant differences between TradAC and ConsAC.

The main advantages that emerge from the analysis of the literature can be summarized in the following points:

✓ Increased fracture resistance: This improves fracture resistance, thereby reducing the risk of post‐endodontic fractures.
✓ Improved mechanical stability of the teeth compared to traditional AC openings.
✓ Long‐term functional survival superior to TradAC.
✓ Direct access to canal orifices: Techniques such as TrussAC and CBCT‐guided access provide direct access to canal orifices.
✓ Greater preservation of precervical and root canal dentin.
✓ Post‐endodontic restorations are less complex to perform with less demolitive restorations.
✓ Cost reduction: Reducing the complexity and destructiveness of restorations leads to cost reduction.

From a clinical and practical standpoint, it is evident that traditional openings, which are less dependent on the operator, certainly lead to better removal of debris and pulp residues, with a greater localization of canal orifices. However, this occurs at the expense of greater consumption of healthy enamel–dentin tissue. In contrast, conservative methods, with the use of guided techniques, still represent a valid alternative, considering the preservation of healthy tissue that could contribute to the success of the restoration.

Divergent results among different studies could stem from the heterogeneity of the tested samples, with some studies consisting only of maxillary molars and others only of mandibular molars, making it futile to aggregate the results in a meta‐analysis. Furthermore, the excessive heterogeneity in study designs renders additional analyses aimed at aggregating data from different studies inconsistent.

In some cases, as highlighted in the observational study by Buchgreitz, Buchgreitz and Bjørndal (2019), despite adherence to STROBE guidelines, the treated teeth were not specified. The report only provides the number of mandibular and maxillary teeth, lacking specific indications regarding their anterior or posterior position. This lack of detail places these studies at risk of bias, potentially influencing the assessment of evidence quality using the GRADE system.

Moreover, the possibility of exclusively focusing on studies addressing anterior or posterior teeth and further excluding in vitro and resin model studies would have certainly eliminated heterogeneity. However, this would have resulted in the inclusion of a lower number of studies, less than 4, and this goes beyond the objectives of the research question in this scoping review.

Despite advances in ConsAC design, there remains a significant gap in the literature regarding specific ACs used in vital pulp therapy (VPT) procedures, such as cervical pulpotomy and partial pulpotomy (Guan et al. 2021). These procedures aim to preserve as much healthy tooth structure as possible while effectively managing pulp health. The relationship between AC design and VPT success rates remains an underexplored area.

VPT seeks to conservatively manage deep carious lesions and exposed pulp. Recently, VPT has evolved with the use of biomaterials such as tricalcium silicate, CBCT (Okamoto et al. 2018), and dental operative microscopy, as well as a better understanding of pulp repair mechanisms. Documented case reports indicate that nonselective carious tissue removal can reduce pulp exposure (Motoki et al. 2021), preserving tooth vitality and promoting the formation of reparative dentine.

In the literature, data on ACs for partial and cervical pulpotomy procedures are not definitive. Some argue that leaving carious tissue might support pulp inflammation, leading to VPT failure. Purulent secretion from exposed pulp is generally seen as a sign of irreversible damage, necessitating pulpectomy (Uesrichai et al. 2019). A recent case published by Okamoto et al. in 2018 discussed the effective VPT procedure on a symptomatic molar with deep caries and purulent pulp secretion (Okamoto et al. 2018). These findings raise questions, given the controversial nature of the topic, about the appropriateness of leaving infected dentine to avoid pulp exposure and whether the presence of purulent pulp tissue necessitates pulpectomy. Thus, although VPT is an increasingly widespread practice, there is a lack of detailed literature analysis on how cavity preparation technique and size may influence treatment success, especially with regard to the risk of leaving infected dentine or debris, and on the importance of accurate intraoperative diagnosis of vital or infected pulp tissue using tools such as the operating microscope. This prompts further investigation into whether a balanced approach in carious cavity openings, with potential pulp exposure and access to the pulp chamber, is essential for preserving tooth structure while ensuring tissue disinfection and long‐term success.

### 4.4 Limitations of the Review

The limitations of this review can be attributed to the limited number of included studies and the heterogeneity of the data, making it impractical to conduct a meta‐analysis. Furthermore, the significant variation in nomenclature for the names of cavity access designs also complicates the identification and categorization of a specific design into a particular group.

The limitations inherent in this scoping review still render the findings provisional and inconclusive regarding the diverse impacts of cavity access designs on the localization of canal orifices and the quantity and presence of debris and pulp residues. A discernible data trend suggests that traditional openings facilitate easier localization of orifices and aid in the removal of debris and pulp residues.

### 4.5 Future Research

Conducting a systematic review, potentially including a meta‐analysis, may be necessary if a sufficient number of suitable clinical studies exist and if there are controversies on the subject. However, given the current scientific literature, a systematic review focusing on specific outcomes, such as the localization of canal orifices and the presence of remaining pulp residues and debris, could only be performed on in vitro studies. This would not be registered on the PROSPERO platform. Therefore, a scoping review or a mapping review is deemed more appropriate while still maintaining methodological rigor.

Future research must delve more comprehensively into aspects related to minimally invasive ACs and how these factors may influence the long‐term success of endodontic treatment. Although the localization of canal orifices represents one of the initial phases of treatment, it is imperative to note that inadequately performed openings could have consequences even in the canal shaping phase. This increases the risk of apical foramen transportation, root stripping, and fracture of rotary endodontic instruments.

Specifically, several points can be expanded upon and form the basis for future research directions to fill gaps in the literature. Retrospective studies are needed, but more importantly, clinical trials with extensive follow‐ups are crucial. Additionally, studies aimed at optimizing techniques to implement minimally invasive AC procedures more predictable are essential. The goal is to find an optimal balance between preserving tooth structure, ensuring accurate disinfection, and achieving proper endodontic filling, ultimately leading to long‐term success.

Diagnostic methods can be further enhanced, such as the use of CBCT, to improve the accuracy of canal orifice identification and reduce procedural errors. This should include evaluations of the cost–benefit ratio of implementing such technologies in routine clinical practice.

Furthermore, biomechanical analysis of treated teeth, using finite element analysis with different AC designs, can provide insights into how the design of the AC impacts the strength and overall mechanical properties of endodontically treated teeth.

In the short term, to validate the efficacy of minimally invasive access methods, it is necessary to conduct clinical trials, including longitudinal studies that evaluate the long‐term outcomes of different AC designs, focusing on factors such as fracture resistance and the survival rates of teeth post‐endodontic treatment.

Comparative efficacy studies involving large, stratified patient populations to assess the relative failure and success rates of TradAC versus ConsAC (UltraAC, TrussAC) should include long‐term follow‐ups and aim for greater standardization of methodologies to increase the robustness of the conclusions.

## 5 Conclusions

In conclusion, this scoping review aimed to comprehensively summarize the existing literature on the influence of AC design on the identification of canal orifices and the presence of pulp residues and debris during endodontic procedures. Although the results provide valuable insights, further research is needed to establish more conclusive evidence in this field.

Study outcomes are inconsistent and sometimes inconclusive. There is a trend indicating that conservative techniques tend to produce more debris and pulp residues, whereas guided techniques show greater precision in locating endodontic canal openings.

The choice of AC design remains a critical consideration for endodontic practitioners, especially when dealing with challenging canal identification and preventing the accumulation of pulp and dentinal residues.

## Author Contributions

Conceptualization: Mario Dioguardi and Davide La Notte. Methodology: Mario Dioguardi and Diego Sovereto. Software: Mario Dioguardi and Cristian Quarta. Formal analysis: Mario Dioguardi and Andrea Ballini. Investigation: Mario Dioguardi and Riccardo Aiuto. Data curation: Mario Dioguardi and Mario Alovisi. Writing–original draft preparation: Mario Dioguardi and Davide La Notte. Writing–review and editing: Mario Dioguardi and Andrea Ballini. Visualization: Mario Dioguardi, Cristian Quarta, and Diego Sovereto. Bioinformatic analysis review: Angelo Martella. Supervision: Lorenzo Lo Muzio and Vito Crincoli.

## Conflicts of Interest

The authors declare no conflicts of interest.

## Data Availability

Data sharing is not applicable to this article as no new data were created or analyzed in this study.
